# Comparison of different dental age estimation methods with deep learning: Willems, Cameriere-European, London Atlas

**DOI:** 10.1007/s00414-025-03452-y

**Published:** 2025-02-19

**Authors:** Betul Sen Yavuz, Omer Ekmekcioglu, Handan Ankarali

**Affiliations:** 1 Department of Pediatric Dentistry, Faculty of Dentistry, Marmara University, Istanbul, Türkiye; 2 Department of Operational Research and Systems, Business School, University of Warwick, Coventry, UK; 3 Department of Biostatistics and Medical Informatics, Istanbul Medeniyet University Faculty of Medicine, Istanbul, Türkiye

**Keywords:** Dental age estimation, Deep learning, Willems method, Cameriere-European formula, London Atlas

## Abstract

This study aimed to compare dental age estimates using Willems, Cameriere-Europe, London Atlas, and deep learning methods on panoramic radiographs of Turkish children. The dental ages of 1169 children (613 girls, 556 boys) who agreed to participate in the study were determined by 4 different methods. The Convolutional Neural Network models examined were implemented in the TensorFlow library. Simple correlations and intraclass correlations between children’s chronological ages and dental age estimates were calculated. Goodness-of-fit criteria were calculated based on the errors in dental age estimates and the smallest possible values for the Akaike Information Criterion, the Bayesian-Schwarz Criterion, the Root Mean Squared Error, and the coefficient of determination value. Simple correlations were observed between dental age and chronological ages in all four methods (*p* < 0.001). However, there was a statistically significant difference between the average dental age estimates of methods other than the London Atlas for boys (*p* = 0.179) and the four methods for girls (*p* < 0.001). The intra-class correlation between chronological age and methods was examined, and almost perfect agreement was observed in all methods. Moreover, the predictions of all methods were similar to each other in each gender and overall (Intraclass correlation [ICC_W_] = 0.92, ICC_CE_=0.94, ICC_LA_=0.95, ICC_DL_=0.89 for all children). The London Atlas is only suitable for boys in predicting the age of Turkish children, Willems, Cameriere-Europe formulas, and deep learning methods need revision.

## Clinical trial registration

The present study is not a clinical trial.

## Introduction

Dental age determination plays a significant role in forensic and clinical dentistry, particularly in the fields of pediatric dentistry and orthodontics [1]. Various methods are being investigated to estimate dental age, especially in children and adolescents. Determining dental age is crucial for timing appropriate treatment. Medical techniques developed based on age include the analysis of bone maturity and dental development. However, chronic illnesses or nutritional deficiencies during an individual’s growth and development can lead to deviations in age estimation. Dental development is more closely related to genetic factors than environmental factors relative to skeletal maturity [2].

Different dental age estimation methods calculate dental age based on criteria such as tooth calcification and eruption levels using dental radiographs [3–5]. The Willems formula modified Demirjian’s method to calculate dental age for the 7 teeth in the left mandible by scoring each tooth according to its level of calcification [5]. The Cameriere-European method includes a regression formula that normalizes data by dividing the distance of the apical opening by tooth length [4]. The London Atlas consists of images showing tooth eruption and calcification levels at one-year intervals up to the age of 15.5 [3]. While the Willems method is developed for estimating dental age in White Belgians [5] and the Cameriere-European method for European populations [4], the schematic images in the London Atlas are created by examining skeletal remains in the United Kingdom [3]. Although some methods for dental age estimation are considered better than others in the literature, none have been reported to accurately predict the age of Turkish children living in Turkey. Although some methods for dental age estimation are considered superior in the literature, specific methodologies providing accurate results for Turkish children have not been identified and/or developed [1, 6, 7].

Currently, deep learning provides satisfactory results in estimating bone age, and its use for dental age determination is increasing. Deep learning, a fundamental approach in artificial intelligence, efficiently and accurately estimates dental age. Deep learning methods have been found to be more successful than traditional radiographic methods [2, 8, 9]. Additionally, the rapid results provided by deep learning in dental age estimation have significantly shortened the procedure compared to manual measurements from radiographs [10].

There is no study in the literature that assesses the dental age of the Turkish population using deep learning. This study aims to evaluate the success of three different methods and a deep learning method based on dental radiographs in predicting the dental age of the Turkish population. The null hypothesis was that each method would produce similar results in estimating dental age.

## Materials and methods

### Subjects

The study protocol was assessed and approved by the Clinical Ethics Committee of Marmara University with approval number 05.05.2023.720. The study was conducted in accordance with the principles of medical research involving human subjects stated in the Declaration of Helsinki. Written informed consent was obtained from the parents or legal guardians of all subjects in the study.

This study was conducted by analyzing digital panoramic radiographs of children aged 5 to 16 years who were admitted to Marmara University, Faculty of Dentistry Clinics for routine dental care between May 2023 and September 2023. Patients at Marmara University consist of people from northwestern Turkey, living in Istanbul or the surrounding provinces. Based on the study of Tural [11], the sample size with G*Power 3.1 software (Faul, Erdfelder, Lang, and Buchner, Düsseldorf, Germany) was found to be 773 with 95% confidence (1-α), 95% test power (1-β), 0.130 effect size. Taking into account the approximately 20% attrition, the study population was determined as 930.

### Selection criteria

Children eligible for inclusion in the study are those who had panoramic radiographs taken between May 2023 and July 2024, were aged between 5 and 16 at the time of radiographic imaging, had radiographs deemed diagnostically excellent (Grade 1) according to the United Kingdom National Radiological Protection Board [12], and did not have any systemic diseases or syndromes.

Children with hyperdontia, hypodontia, or any dental anomalies, periapical or periodontal lesions, any form of tooth loss for any reason, a history of orthodontic treatment, jawbone cysts and tumors, or a history of orofacial and dental trauma will be excluded from the study.

### Radiographic evaluation

**Fig. 1:**
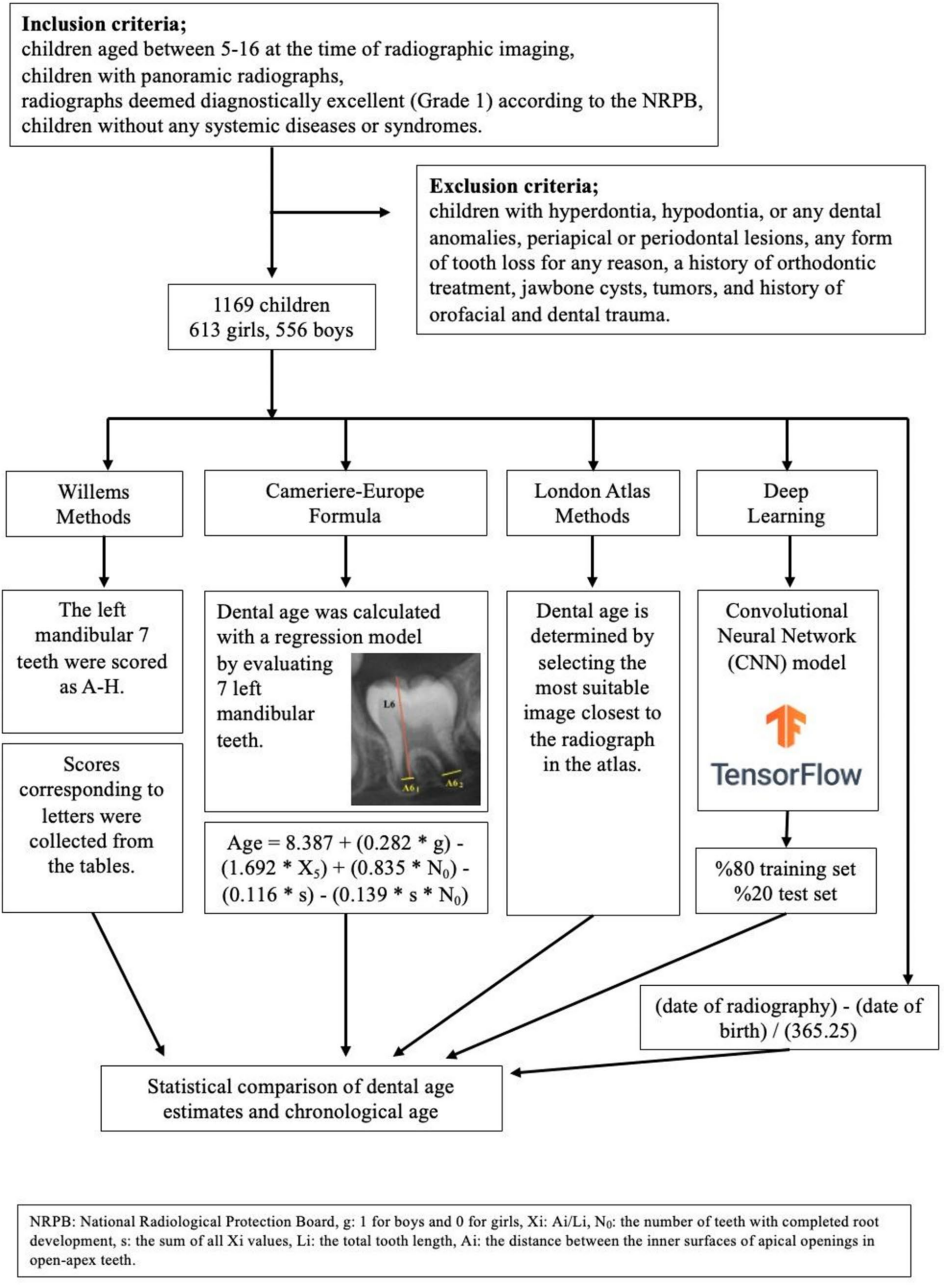
The flowchart of patient enrollment and data analysis

All the digital panoramic radiographs included in the study were acquired using a Morita device (VeraView IC5, J. Morita MFG. Corporation, Kyoto, Japan) with an exposure time of 8.8 s, a power of 60–70 kV, and a current of 7.5 mA at the Oral Diagnosis and Radiology Clinic of Marmara University Faculty of Dentistry. The diagnostic accuracy of the radiographs was assessed in accordance with the criteria of the United Kingdom National Radiological Protection Board [12]. According to these criteria, Grade 1 represents diagnostically excellent radiographs without any irradiation, positioning, or procedural errors, while Grade 2 radiographs may include some minor irradiation, positioning, or procedural errors. Grade 3 radiographs, on the other hand, contain irradiation, positioning, and/or procedural errors to an extent that is deemed unacceptable in the evaluation. In terms of the validity of assessments, it has been stipulated that at least 70% of the radiographs included in a study should be Grade 1, less than 20% should be Grade 2, and less than 10% should be Grade 3 [12]. In this study, only panoramic radiographs of Grade 1 diagnostic quality were considered for evaluation. The images were labeled and saved with the child’s date of birth and the date of the panoramic radiograph. The flowchart of patient enrollment and data analysis is shown in Fig. 1.

### Chronological age calculation

The chronological age of patients will be calculated in decimal form using the formula [(date of panoramic radiograph) - (official date of birth)] / 365.25 in Microsoft Excel software. Patient gender information will also be recorded alongside their chronological ages.

### Age estimation of radiographic methods using developing teeth

Dental age determination will be performed using the Willems, Cameriere-European, and London Atlas methods.

### Willems

The Willems method was developed using the ANOVA test by Willems et al. [5] as an improvement to age determinations based on the Demirjian method, which tended to overestimate age when compared to chronological age. This method was initially applied to the white Belgian population. Age determination using the Willems method relies on the dental mineralization stages of the left mandibular 7 molars. These stages are scored based on tables provided by Willems, and the sum of the scores directly provides the individual’s dental age [5].

### Cameriere-European

The Cameriere-European formula was developed in 2007 by Cameriere and colleagues for dental age determination in children from Europe and surrounding countries. This method involves the measurement of root development and apical opening in the lower left mandibular teeth. The number of teeth with completed root development is represented as N_0_. The distance between the inner surfaces of apical openings in single-rooted (Ai, i = 1,.,5) and multi-rooted (Ai, i = 6,7) open-apex teeth was measured. To minimize potential magnification and angulation errors, the designated distance for each tooth was divided (Xi) by the total tooth length (Li, i = 1,.,7). The sum of all Xi values, along with the s value (total Ai/Li values), is inserted into the Cameriere-European formula to calculate the individual’s dental age. In the formula, the variable g is assigned as 1 for boys and 0 for girls, and the formula is expressed as “Age = 8.387 + (0.282 * g) - (1.692 * X_5_) + (0.835 * N_0_) - (0.116 * s) - (0.139 * s * N_0_)” [4, 13].

### London Atlas

The London Atlas, developed in 2010, consists of a series of schematic images created for specific ages. Based on evidence and available in multiple languages, the reference images in the atlas are examined to determine the most suitable image for a child’s panoramic radiograph [1, 3].

For measurements, all radiographs included in the study were transferred to a computer-assisted measurement program (ImageJ version 1.49v, National Institutes of Health, Bethesda, Maryland, USA). All manual methods were measured by two different pediatric dentists with 7 and 9 years of experience.

### Deep learning algorithm

The performance of deep learning methods, specifically Convolutional Neural Networks (CNNs) for dental age estimation was assessed. To conduct the experiments, the dataset was split into two: training and testing using the common split scheme of 80 − 20%. Furthermore, the training data was further divided into training and validation sets to perform hyperparameter tuning and select the model architecture, with 90% of the data used for training and the remaining 10% for validation. Final estimates corresponding to the t-test data were reported. This experiment was performed in the spirit of cross-validation five times to predict the whole dataset and obtain comparable results with the other examined methods.

**Fig. 2:**
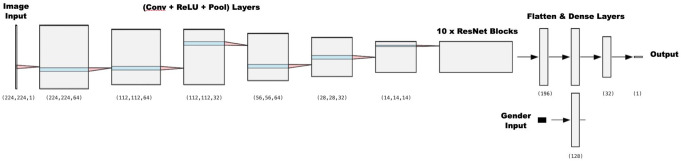
CNN model architecture used for Deep Learning analysis

In the preparation of the dataset, X-ray images were paired with the chronological ages and gender information of the patients. The images were downsized to reduce the computational load of the training process. A resolution of 224 × 224 pixels was selected for the downsized images. In addition to the X-ray images, the gender information of each patient was incorporated as an additional feature to improve the accuracy of the estimation results (Fig. 2**)**.

In this study, various architectures commonly used in deep learning, including ResNet, Xception, EfficientNet, and similar CNN models, were evaluated. Furthermore, transfer learning was examined to determine if it contributed to obtaining better results. It was observed that transfer learning using a pre-trained model, such as ResNet50, not only failed to improve the results but also slightly degraded performance, likely due to the limited number of training examples available for fine-tuning. As a result, a simpler ResNet model is used. The model consists of four convolutional layers. In the first convolutional layer, we used a filter size of 64 and a kernel size of 3 × 3 and applied max pooling operation with a 2 × 2 pooling window. Subsequently, the same convolution and max pooling operations were repeated three times, with the only difference being a reduction in the number of filters to 32 in the convolutional layers. These convolutions were followed by ten layers of ResNet blocks. After flattening the output of the ResNet blocks, five feed-forward layers were used to obtain the chronological age prediction. All models were implemented using the TensorFlow library [14].

### Statistical analyses

For the Willems, Cameriere-European, London Atlas methods, 200 randomly selected radiographs were re-evaluated at two-week intervals and intra- and inter-examiner agreement levels were assessed using Intraclass Correlation Coefficients (ICC). The distribution of age categories by gender was analyzed using the Chi-Square (χ²) test.

The Kolmogorov-Smirnov test was used to examine whether the ages had a normal distribution. Gender differences regarding age were determined using the Independent Samples t-test. Paired Samples t-test was used for testing differences between chronological age, and the predicted dental age obtained from four methods. Relationships between predicted and chronological ages were preliminarily examined using a Pearson Correlation analysis. The agreement between the chronological age and the dental age was investigated by using the Intraclass Correlation Coefficient. The ICC was interpreted according to the categories suggested by Shrout and Fleiss [15]. Additionally, the performance of predicting chronological age using the deep learning algorithm was evaluated with various goodness of fit criteria (Mean Squared Error, AIC, etc.).

The chronological ages of the patients and the predicted dental ages using four different methods were compared for each age group using Paired Samples t-tests for normally distributed data and Wilcoxon Signed-Rank tests for non-normally distributed data. The mean differences between the chronological ages and the predicted ages were calculated for each age group. Additionally, mean absolute errors (MAE) were computed to determine the accuracy of the estimation methods. Analyses were performed using SPSS (Ver. 26.0) and R 4.2.2 software. A statistical significance level was accepted as 0.05.

## Results

The study included a total of 1169 children, with chronological ages ranging from 5 to 16, including 613 girls and 556 boys. For each age within the given age range, there were at least 27 and at most 73 individuals. Gender distribution according to age groups is given in Table 1. There was no statistically significant difference in the distribution of age categories between genders (χ²=16.575, *p* = 0.121).

**Table 1:** Gender distribution according to age groups

Age groups	Gender		
	Girl	Boy	Total (%)
5.00–5.99	35 (5.71)	47 (8.45)	82 (7.02)
6.00–6.99	58 (9.46)	45 (8.09)	103 (8.81)
7.00–7.99	44 (7.18)	58 (10.43)	102 (8.73)
8.00–8.99	59 (9.63)	56 (10.07)	115 (9.84)
9.00–9.99	68 (11.09)	60 (10.79)	128 (10.95)
10.00–10.99	60 (9.79)	73 (13.13)	133 (11.38)
11.00–11.99	58 (9.46)	53 (9.53)	111 (9.50)
12.00–12.99	68 (11.09)	57 (10.25)	125 (10.69)
13.00–13.99	60 (9.79)	44 (7.91)	104 (8.90)
14.00–14.99	51 (8.32)	35 (6.30)	86 (7.36)
15.00–15.99	52 (8.48)	28 (5.04)	80 (6.84)
Total (%)	613 (100)	556 (100)	1169 (100)

The ICC values demonstrated excellent reliability and consistency across all three methods. For the Willems method, intra-examiner reliability was 0.98 and 0.95, while inter-examiner reliability was 0.85. For the Cameriere-European method, intra-examiner reliability was 0.95 and 0.94, while inter-examiner reliability was 0.87. For the London Atlas method, intra-examiner reliability was 0.89 and 0.90, while inter-examiner reliability was 0.84. All ICC values ranged from 0.81 to 0.99, indicating almost perfect reliability and agreement between and among examiners according to Shrout and Fleiss [15]. No statistically significant differences were observed between methods or examiners, supporting these assessment techniques’ robustness.

Additionally, considering the differences in the development of girls and boys, dental age was separately estimated for girls and boys according to three methods, and new predictions were also made using a deep learning algorithm. The results presented in Table 2 were obtained by comparing the mean of these predictions with the mean of the chronological age. When Table 2 was evaluated, there was no significant difference between the mean prediction made by the London Atlas and the mean of chronological age in boys, while the predictions of the other two methods and the deep learning algorithm differ significantly from the mean of chronological age. The Cameriere-European formula and deep learning underestimated the dental age of all children with a mean of 0.35 and 0.09 years, respectively, while the Willems and London Atlas methods overestimated the dental age with a mean of 0.68 and 0.16 years, respectively. Furthermore, there was a significant and high degree of correlation between the predictions of deep learning as well as the known three methods and chronological age in both girls and boys (Table 2).

**Table 2:** Comparative results and correlations of chronological age and predicted dental age by four methods

Gender	Mean difference between chronological age and each method prediction				Simple correlation between chronological age and each method prediction	
	Chronological age	Methods	Predicted dental age			
	Mean ± SD		Mean ± SD	P^a^	r	P^b^
Girls (*n* = 613)	10.69 ± 2.97	**W**	11.37 ± 3.35	**< 0.001**	0.944	**< 0.001**
		**CE**	10.30 ± 2.60	**< 0.001**	0.951	**< 0.001**
		**LA**	10.95 ± 2.91	**< 0.001**	0.954	**< 0.001**
		**DL**	10.61 ± 2.48	**< 0.001**	0.810	**< 0.001**
Boys (*n* = 556)	10.17 ± 2.87	**W**	10.85 ± 2.99	**< 0.001**	0.952	**< 0.001**
		**CE**	9.86 ± 2.54	**< 0.001**	0.949	**< 0.001**
		**LA**	10.22 ± 2.92	0.179	0.948	**< 0.001**
		**DL**	10.06 ± 2.5	**< 0.001**	0.834	**< 0.001**

SD: Standard deviation, W: Willems, CE: Cameriere-Europe, LA: London Atlas, DL: Deep Learning, ^a^: Paired samples t-test, ^b^: Pearson correlation analysis

The agreement between chronological age and dental age predicted by the methods was the measure that best reflects the success of these predictions. For this purpose, ICC coefficients showing the agreement between chronological age and predictions were calculated, and the results are given in Table 3. When the table was examined, all ICC coefficients were above 0.81, indicating an almost perfect fit. Although all methods had quite successful predictions, the deep learning algorithm’s prediction was slightly weaker than the other three methods. In addition, the predictions were the same according to the gender for the London Atlas, Another supporting finding for these results was the goodness-of-fit criteria given in Table 4. These criteria were calculated based on the errors in these predictions, and the smallest possible values for AIC (Akaike Information Criterion), BIC (Bayesian-Schwarz Criterion), and RMSE (Root Mean Squared Error) criteria, and an R-square (Determination coefficient) value close to 100%, indicate the best predictions. In this respect, it was seen that the predictions of the three methods were almost similar to each other, but the predictions of deep learning were less successful. When these criteria are examined, it was observed that the AIC, BIC, and RMSE values were the lowest for the London Atlas method, followed by the Cameriere-Europe, Willems, and Deep Learning methods, respectively. A lower AIC indicates a better balance between the model’s fit quality and complexity, while a lower BIC signifies that the model is more suitable for the dataset and less complex. When RMSE values were analyzed, an approximate deviation of 0.9 in dental age was observed for the three manual methods, whereas this deviation was approximately 1.7 in the Deep Learning method.

**Table 3:** The agreement between the chronological age and the dental age according to the dental age estimation methods in each gender

	Agreement							
	CA and W		CA and CE		CA and LA		CA and DL	
	ICC	95% CI	ICC	95% CI	ICC	95% CI	ICC	95% CI
Girls	0.92	0.90–0.93	0.93	0.92–0.94	0.95	0.94–0.96	0.88	0.87–0.90
Boys	0.93	0.91–0.94	0.94	0.92–0.95	0.95	0.94–0.96	0.90	0.88 − 0.68
Total	0.92	0.91–0.93	0.94	0.93–0.94	0.95	0.94–0.96	0.89	0.88–0.91

ICC: Intraclass correlation coefficient, CA: Chronological age (gold standard), W: Willems, CE: Cameriere-Europe, LA: London Atlas, DL: Deep Learning, CI: Confidence Interval

**Table 4:** Model goodness of fit measures

Method	AIC	BIC	RMSE	*R* ^2^
Willems	3176	3192	0.939	0.897
Cameriere-Europe	3115	3130	0.915	0.903
London Atlas	3096	3111	0.907	0.904
Deep Learning	4519	4535	1.670	0.676

AIC: Akaike Criterion, BIC: Bayesian-Schwarz Criterion, RMSE: Root Mean Squared Error, R^2^: Determination coefficient

Table 5 (for girls) and Table 6 (for boys) presents the means and standard deviations of dental ages calculated using each method, the differences between dental and chronological ages, MAE, and chronological ages, and p-values for each age group in girls. For girls, the Willems method overestimated the chronological age within a range of 0.20 to 1.30. The Cameriere-European method showed a wider variation, overestimating up to 0.91 and underestimating up to 1.65. The London Atlas method overestimated up to 0.80 and underestimated up to 0.41, while the Deep Learning method demonstrated the largest range, overestimating up to 1.38 and underestimating up to 2.45. In boys, the Willems method showed overestimations ranging from 0.06 to 1.00. The Cameriere-European method had overestimations reaching 0.91 and underestimations extending to 1.45. The London Atlas method produced smaller deviations, overestimating by up to 0.29 and underestimating by 0.52. Lastly, the Deep Learning method displayed the most extensive range, with overestimations as high as 1.56 and underestimations as low as 1.95.

**Table 5:** Comparison of dental age predicted by different dental age estimation methods and chronological age in all age groups of girls

Age	*N* (%)	Chronological age (Mean ± SD)	Methods	Predicted dental age (Mean ± SD)	Difference (Mean ± SD)	MAE (Mean ± SD)	*P*
5.00–5.99	35 (5.71)	5.58 ± 0.29	**W**	5.77 ± 0.79	-0.20 ± 0.60	0.54 ± 0.32	0.060^a^
			**CE**	6.48 ± 0.33	-0.91 ± 0.31	0.91 ± 0.31	**< 0.001** ^**a**^
			**LA**	5.96 ± 0.51	-0.38 ± 0.44	0.49 ± 0.31	**< 0.001** ^**b**^
			**DL**	6.83 ± 0.87	-1.25 ± 0.95	1.26 ± 0.93	**< 0.001** ^**a**^
6.00–6.99	58 (9.46)	6.60 ± 0.30	**W**	6.94 ± 0.81	-0.35 ± 0.73	0.63 ± 0.50	**< 0.001** ^**b**^
			**CE**	6.70 ± 0.67	-0.10 ± 0.71	0.39 ± 0.60	**0.001** ^**b**^
			**LA**	6.71 ± 0.70	-0.11 ± 0.55	0.49 ± 0.28	0.094^b^
			**DL**	7.98 ± 1.36	-1.38 ± 1.35	1.49 ± 1.23	**< 0.001** ^**b**^
7.00–7.99	44 (7.18)	7.49 ± 0.29	**W**	8.36 ± 0.65	-0.87 ± 0.56	0.91 ± 0.49	**< 0.001** ^**b**^
			**CE**	7.28 ± 0.55	0.21 ± 0.49	0.43 ± 0.30	**0.004** ^**b**^
			**LA**	7.66 ± 0.65	-0.17 ± 0.56	0.48 ± 0.33	**0.026** ^**b**^
			**DL**	7.93 ± 1.14	-0.44 ± 1.13	0.88 ± 0.83	**0.013** ^**a**^
8.00–8.99	59 (9.63)	8.53 ± 0.28	**W**	9.18 ± 0.28	-0.64 ± 0.56	0.71 ± 0.47	**< 0.001** ^**b**^
			**CE**	8.28 ± 0.82	0.25 ± 0.80	0.69 ± 0.47	**0.019** ^**a**^
			**LA**	9.33 ± 0.89	-0.80 ± 0.88	0.92 ± 0.74	**< 0.001** ^**b**^
			**DL**	9.08 ± 1.73	-0.55 ± 1.77	1.29 ± 1.32	**0.044** ^**b**^
9.00–9.99	68 (11.09)	9.53 ± 0.32	**W**	9.94 ± 0.90	-0.41 ± 0.85	0.65 ± 0.68	**< 0.001** ^**b**^
			**CE**	9.36 ± 0.96	0.17 ± 0.86	0.62 ± 0.61	0.097^b^
			**LA**	10.10 ± 1.16	-0.58 ± 0.1.10	0.96 ± 0.78	**< 0.001** ^**b**^
			**DL**	9.87 ± 1.39	-0.34 ± 1.37	1.07 ± 0.92	**0.042** ^**b**^
10.00–10.99	60 (9.79)	10.46 ± 0.29	**W**	10.66 ± 0.95	-0.20 ± 0.86	0.72 ± 0.50	0.102^b^
			**CE**	10.17 ± 0.76	0.30 ± 0.70	0.61 ± 0.46	**0.001** ^**b**^
			**LA**	11.00 ± 1.02	-0.54 ± 0.95	0.96 ± 0.51	**< 0.001** ^**b**^
			**DL**	10.33 ± 1.62	0.13 ± 1.54	1.21 ± 0.95	0.444^b^
11.00–11.99	58 (9.46)	11.47 ± 0.27	**W**	12.19 ± 1.28	-0.72 ± 1.29	1.09 ± 0.99	**0.001** ^**b**^
			**CE**	11.15 ± 0.86	0.32 ± 0.85	0.77 ± 0.48	**0.007** ^**b**^
			**LA**	11.83 ± 0.80	-0.36 ± 0.82	0.73 ± 0.50	**< 0.001** ^**b**^
			**DL**	11.80 ± 1.49	-0.33 ± 1.45	1.18 ± 0.90	0.088^a^
12.00–12.99	68 (11.09)	12.45 ± 0.31	**W**	13.63 ± 0.31	-1.18 ± 1.51	1.52 ± 1.17	**< 0.001** ^**b**^
			**CE**	13.63 ± 1.61	0.31 ± 0.92	0.80 ± 0.55	**0.014** ^**b**^
			**LA**	12.14 ± 1.01	-0.34 ± 0.94	0.86 ± 0.50	**0.011** ^**b**^
			**DL**	12.79 ± 1.07	0.29 ± 1.40	1.19 ± 0.79	0.128^b^
13.00–13.99	60 (9.79)	13.51 ± 0.28	**W**	14.81 ± 1.57	-1.30 ± 1.51	1.74 ± 0.96	**< 0.001** ^**b**^
			**CE**	12.81 ± 0.89	0.69 ± 0.87	0.78 ± 0.79	**< 0.001** ^**b**^
			**LA**	13.48 ± 0.91	0.02 ± 0.89	0.65 ± 0.60	0.517^b^
			**DL**	12.94 ± 1.43	0.57 ± 1.41	1.12 ± 1.03	**0.009** ^**b**^
14.00–14.99	51 (8.32)	14.47 ± 0.26	**W**	15.39 ± 1.33	-0.93 ± 1.30	1.30 ± 0.91	**< 0.001** ^**b**^
			**CE**	13.36 ± 0.78	1.11 ± 0.71	1.11 ± 0.71	**< 0.001** ^**b**^
			**LA**	14.28 ± 0.95	0.18 ± 0.87	0.78 ± 0.40	0.183^b^
			**DL**	12.95 ± 1.22	1.51 ± 1.26	1.60 ± 1.15	**< 0.001** ^**a**^
15.00–15.99	52 (8.48)	15.49 ± 0.30	**W**	16.00 ± 0.30	-0.51 ± 1.07	1.07 ± 0.50	**0.001** ^**b**^
			**CE**	13.84 ± 0.73	1.65 ± 0.77	1.65 ± 0.77	**< 0.001** ^**b**^
			**LA**	15.08 ± 0.70	0.41 ± 0.74	0.59 ± 0.60	**0.001** ^**b**^
			**DL**	13.04 ± 1.52	2.45 ± 1.52	2.45 ± 1.52	**< 0.001** ^**b**^

SD: Standard deviation, MAE: Mean absolute error, Difference: Chronological age – Predicted dental age,

W: Willems, CE: Cameriere-Europe, LA: London Atlas, DL: Deep Learning, ^a^: Paired samples t-test,

^b^: Wilcoxon signed-rank test

**Table 6:** Comparison of dental age predicted by different dental age estimation methods and chronological age in all age groups of boys

Age	*N* (%)	Chronological age (Mean ± SD)	Methods	Predicted dental age (Mean ± SD)	Difference (Mean ± SD)	MAE (Mean ± SD)	*P*
5.00–5.99	47 (8.45)	5.57 ± 0.28	**W**	5.98 ± 0.74	-0.41 ± 0.57	0.55 ± 0.43	**< 0.001** ^**b**^
			**CE**	6.48 ± 0.37	-0.91 ± 0.44	0.91 ± 0.44	**< 0.001** ^**b**^
			**LA**	5.86 ± 0.49	-0.29 ± 0.48	0.44 ± 0.36	**0.001** ^**b**^
			**DL**	7.13 ± 1.05	-1.56 ± 1.05	1.59 ± 1.01	**< 0.001** ^**b**^
6.00–6.99	45 (8.09)	6.52 ± 0.25	**W**	7.13 ± 0.61	-0.61 ± 0.55	0.70 ± 0.43	**< 0.001** ^**a**^
			**CE**	6.86 ± 0.32	-0.34 ± 0.39	0.44 ± 0.27	**< 0.001** ^**a**^
			**LA**	6.39 ± 0.61	0.13 ± 0.60	0.47 ± 0.39	0.225
			**DL**	7.36 ± 0.96	-0.84 ± 0.89	0.96 ± 0.75	**< 0.001** ^**a**^
7.00–7.99	58 (10.43)	7.45 ± 0.31	**W**	8.24 ± 0.66	-0.78 ± 0.64	0.89 ± 0.47	**< 0.001** ^**b**^
			**CE**	7.22 ± 0.43	0.24 ± 0.47	0.41 ± 0.33	**< 0.001** ^**b**^
			**LA**	7.31 ± 0.63	0.14 ± 0.58	0.47 ± 0.36	0.142^b^
			**DL**	8.05 ± 1.38	-0.60 ± 1.34	1.01 ± 1.06	**0.003** ^**b**^
8.00–8.99	56 (10.07)	8.52 ± 0.28	**W**	9.38 ± 0.73	-0.86 ± 0.68	0.93 ± 0.59	**< 0.001** ^**a**^
			**CE**	8.09 ± 1.01	0.43 ± 1.00	0.86 ± 0.66	**0.002** ^**b**^
			**LA**	8.70 ± 0.77	-0.18 ± 0.69	0.50 ± 0.50	0.115
			**DL**	8.43 ± 1.25	0.09 ± 1.22	0.94 ± 0.77	0.584^a^
9.00–9.99	60 (10.79)	9.44 ± 0.30	**W**	10.38 ± 0.80	-0.94 ± 0.82	1.04 ± 0.69	**< 0.001** ^**b**^
			**CE**	9.25 ± 0.95	0.19 ± 0.90	0.76 ± 0.50	0.100^a^
			**LA**	9.90 ± 1.36	-0.46 ± 1.35	1.18 ± 0.78	**0.019** ^**b**^
			**DL**	9.29 ± 1.64	0.25 ± 1.59	1.28 ± 0.96	0.236^a^
10.00–10.99	73 (13.13)	10.53 ± 0.27	**W**	11.04 ± 0.87	-0.52 ± 0.79	0.70 ± 0.64	**< 0.001** ^**b**^
			**CE**	10.21 ± 0.54	0.31 ± 0.49	0.44 ± 0.38	**< 0.001** ^**a**^
			**LA**	10.54 ± 1.15	-0.02 ± 1.09	0.94 ± 0.54	0.954
			**DL**	10.34 ± 1.53	0.18 ± 1.48	1.19 ± 0.88	0.299^a^
11.00–11.99	53 (9.53)	11.51 ± 0.31	**W**	12.18 ± 1.23	-0.67 ± 1.22	1.00 ± 0.97	**< 0.001** ^**b**^
			**CE**	11.02 ± 0.98	0.49 ± 0.99	0.93 ± 0.57	**< 0.001** ^**b**^
			**LA**	11.84 ± 0.94	-0.33 ± 0.91	0.80 ± 0.54	**0.005** ^**b**^
			**DL**	11.55 ± 1.53	-0.04 ± 1.54	1.20 ± 0.95	0.487^b^
12.00–12.99	57 (10.25)	12.54 ± 0.30	**W**	13.30 ± 1.28	-0.76 ± 1.24	1.12 ± 0.92	**< 0.001** ^**b**^
			**CE**	11.82 ± 1.09	0.72 ± 1.02	1.06 ± 0.65	**< 0.001** ^**b**^
			**LA**	12.47 ± 1.10	0.08 ± 1.04	0.89 ± 0.53	0.682^b^
			**DL**	11.91 ± 1.45	0.63 ± 1.46	1.19 ± 1.04	**0.002** ^**a**^
13.00–13.99	44 (7.91)	13.44 ± 0.29	**W**	14.45 ± 1.21	-1.00 ± 1.09	1.24 ± 0.80	**< 0.001** ^**b**^
			**CE**	12.93 ± 1.04	0.51 ± 0.92	0.78 ± 0.71	**0.003** ^**b**^
			**LA**	13.46 ± 0.91	-0.01 ± 0.86	0.63 ± 0.58	0.294^b^
			**DL**	12.73 ± 1.47	0.71 ± 1.51	1.17 ± 1.18	**0.016** ^**b**^
14.00–14.99	35 (6.30)	14.44 ± 0.31	**W**	15.00 ± 1.04	-0.57 ± 1.07	0.99 ± 0.68	**0.014** ^**b**^
			**CE**	13.63 ± 0.78	0.81 ± 0.75	0.85 ± 0.69	**< 0.001** ^**b**^
			**LA**	14.21 ± 1.02	0.22 ± 0.98	0.82 ± 0.57	0.258^b^
			**DL**	13.00 ± 1.42	1.43 ± 1.41	1.48 ± 1.35	**< 0.001** ^**b**^
15.00–15.99	28 (5.04)	15.45 ± 0.34	**W**	15.51 ± 0.87	-0.06 ± 0.88	0.72 ± 0.48	0.387^b^
			**CE**	14.00 ± 0.76	1.45 ± 0.72	1.45 ± 0.72	**< 0.001** ^**b**^
			**LA**	14.93 ± 0.74	0.52 ± 0.73	0.65 ± 0.62	**< 0.001** ^**b**^
			**DL**	13.50 ± 0.97	1.95 ± 0.97	1.95 ± 0.97	**< 0.001** ^**b**^

SD: Standard deviation, MAE: Mean absolute error, Difference: Chronological age – Predicted dental age,

W: Willems, CE: Cameriere-Europe, LA: London Atlas, DL: Deep Learning, ^a^: Paired samples t-test,

^b^: Wilcoxon signed-rank test

## Discussion

The age estimation is crucial not only in forensic medicine but also in clinical practice when deciding on dental treatments or determining the ages of individuals whose birth dates are not fully known [2, 9]. Among the methods used for age estimation, calculations based on dental development, which is least affected by environmental factors such as endocrine diseases and nutrition, are used [16]. One advantage of methods that estimate dental age through dental development is that they do not compromise material integrity. Additionally, in estimating the dental age of children using dental development, the evaluation of the developmental stage is carried out while mineralization and eruption of teeth are ongoing. Hence, there is another advantage in terms of very low inter-observer error rates [16]. However, since most methods involve multiple stages related to the mineralization or eruption of teeth, they can be time-consuming, posing a disadvantage in terms of clinical applicability [17]. Therefore, this study aimed to evaluate the success of three different dental age estimation methods and a deep learning algorithm in predicting the chronological age of children aged 5 to 16 living in Turkey.

There are numerous studies evaluating methods such as Haavikko, Nolla, Demirjian, Cameiere, Willems, and London Atlas to estimate the dental age of a group of Turkish children in literature [1, 11, 18–20]. Studies assessing the Demirjian method reported its inadequacy for northern [21], northwestern [7], eastern [6], and western [22] Turkish children. In addition, although it was considered suitable for southern Turkish children, it was noted that the method requires revision [18]. In a study evaluating the Nolla method for predicting the dental age of Turkish children, it was reported that the Nolla method is suitable for boys but not for predicting the dental ages of girls, and both genders’ dental maturity is underestimated [23]. Additionally, Kırzıoğlu et al., [19] asserted that the Nolla method, as well as the Haavikko and Demirjian methods, are not suitable for Turkish children. However, they mentioned that the Haavikko method provides results closer to chronological age compared to other methods. Sezer and Çarıkçıoğlu [20] reported that the London Atlas is suitable for predicting the dental age of northwestern Turkish boys but not for girls and or the population as a whole. They also stated that the Haavikko method provides results closer to chronological age than the London Atlas and Cameriere-European methods but concluded that none of these methods are suitable for predicting the dental age of Turkish children. In 2019, Tural observed that the Demirjian and Willems methods overestimate chronological age, the Haavikko and Cameriere methods underestimate it, and among these methods, the Cameriere method provides the closest estimate of dental age [11]. Sezer et al. [1] evaluated the success of the London Atlas, Cameriere-European, and Willems methods in predicting the ages of children with and without molar-incisor hypomineralization. The findings indicated that the London Atlas and Cameriere-European formula provided more accurate predictions in both groups than the Willems method, which was found to be inaccurate in predicting the dental age of children without molar-incisor hypomineralization. Considering these studies, no method has emerged as suitable for predicting chronological age in the Turkish population. Only some methods have been indicated to provide predictions for specific genders in certain regions of Turkey. The limited sample size in these studies may contribute to these results. Therefore, larger sample size studies, as in the current research, may contribute to the literature. In this study, the dental age of Turkish children was manually evaluated by two researchers using the Willems, Cameriere-European, and London Atlas methods based on panoramic radiography. The findings revealed that the London Atlas accurately predicted the dental age for boys, consistent with the results of Sezer and Çarıkçıoğlu’s study in 2022 [20]. Additionally, it was observed in this study that the London Atlas did not accurately predict the dental age for girls and that the other three methods did not accurately predict chronological age for both genders. This result supports the conclusion that there was still no ideal method for predicting the dental age of Turkish children. Therefore, based on this notion, this study aimed to predict the dental age of Turkish children using not only manual methods but also a deep learning algorithm. Accurate estimation of dental age with deep learning algorithms can make a great contribution to the field of forensic and clinical dentistry in terms of both time and objectivity [10].

In this study, the deep learning method was highly correlated with chronological age, similar to the three manual methods, and its ICC was similarly quite successful. These results are clinically below the expected agreement [24]. The sample size ranging from 79 to 133 per age group may be related to this finding. In the literature, the accuracy of most deep learning applications in dentistry was below 90%. To achieve 98% accuracy in anatomical classification using three-dimensional tomography with deep learning, it was reported that a dataset of over 1000 per group is needed, and to increase this accuracy to 99%, a dataset of over 4000 per group is required [24]. In this study, dental age estimation was performed using panoramic radiographs to obtain a larger dataset than routinely used panoramic radiographs in dental practice. Due to differences in image quality and magnifications of panoramic radiographs compared to three-dimensional tomography, lower accuracy is expected. However, since three-dimensional tomographs are often obtained for reasons such as hyperdontia, jawbone cysts, and tumors, most images are not suitable for predicting the dental age of the general population. However, the absence of image variation due to device and exposure characteristics, as data in this study were obtained with the same exposure standards from a single center, is one of the strengths of the study.

Bunyarit et al. [8] determined the dental age of Malaysian Indian children and adolescents using the Chaillet and Demirjian methods. Subsequently, they developed a new prediction model using a neural network based on these scores. They emphasized that these modified scores provided more accurate dental age predictions [8]. On the other hand, Shen et al. [2] determined the dental age of children aged 5–13 using the Cameriere method and then predicted it using random forest, support vector machine, and linear regression models based on these scores. They reported that machine learning models predicted with better accuracy than the Cameriere formula [2]. In the aforementioned studies, dental age estimation involved researchers manually measuring dental features to enhance prediction accuracy by refining age estimation formulas or scores. In contrast, this study directly utilized images as input for a deep learning algorithm, bypassing the time-consuming manual measurement process. However, the relatively inferior performance of the deep learning models compared to traditional methods may be attributed to this approach, as it relies solely on raw image data and the patient’s gender, without incorporating hand-crafted features that require expert knowledge. By focusing on evaluating the end-to-end performance of deep learning models without expert-driven inputs, this study highlights both the potential and limitations of such an approach. This is another of the strengths of this study. However, it should be noted that the potential limitation of this study is the lower sample sizes per group, which may result in lower accuracy of the deep learning algorithm. To improve the deep learning algorithm, it can increase the data set by collecting data for a longer period. Additionally, the higher AIC and BIC values of the deep learning model may be due to its use of a more complex model with more parameters. In complex models, the size of the dataset becomes more critical. However, in this study, the limitation of the small dataset was addressed by ensuring a balanced distribution among labels such as age and gender to improve the model’s learning performance.

In most studies where deep learning is utilized in dentistry, research has been conducted on subjects such as caries diagnosis or identification of anatomical landmarks using intraoral radiographs or cropped panoramic radiographs [25]. Similarly, Kahaki et al. [26], in their study evaluating dental age using panoramic radiography, separated the image to include the first and third molars. Unlike the dental age estimation performed in this study, these tasks are less complex, primarily because they involve binary classification. Furthermore, caries detection relies on pixel densities, whereas age estimation is conducted across the entire panoramic radiograph. This difference results in more predictable behavior for the former problem. Two studies conducted in 2017 and 2023 used the convolutional neural network approach to classify third molars according to developmental stages and reported a 10% increase in classification accuracy and 91–93% accuracy in classification [27, 28]. In addition, in a study that analyzed the entire panoramic radiography, an average absolute error of 4.06 was determined using age convolutional networks for individuals between the ages of 19–90 [29]. In this study, dental age was estimated not only from the developmental stages of one or a few teeth, as in most studies in the literature [27, 28, 30], but also from all teeth in the panoramic radiography. This has reduced the prediction time to a very short period of 1–2 s. Furthermore, in this study, gender information was used as an additional feature to improve the prediction results. A study published by Vila-Blanco et al. [10] reported that DASNet, which added a second CNN path to include gender features, gave more accurate results than DANet, which includes a sequential CNN path. With access to a larger dataset, a more complex model, such as DASNet, could potentially enhance the Deep Learning results we have observed.

In conclusion, when estimating dental age from direct panoramic radiography with the deep learning method, deep learning showed similar accuracy to the Willems, Cameriere-Europe, and London Atlas methods. The algorithm should be improved in further studies by increasing the number of samples per group or pre-processing of images. Although the London Atlas is only suitable for boys in estimating the dental age of Turkish children, it needs revision in terms of the clinical use of Willems, Cameriere-European formulas, and deep learning methods.

## Data Availability

The data used in this study are available upon reasonable request to the corresponding author.

## Abbreviations

ICC: Intraclass correlation
CNN: Convolutional Neural Network
